# Unique natural killer cell subpopulations are associated with exacerbation risk in chronic obstructive pulmonary disease

**DOI:** 10.1038/s41598-020-58326-7

**Published:** 2020-01-27

**Authors:** Andrew R. Osterburg, Laura Lach, Ralph J. Panos, Michael T. Borchers

**Affiliations:** 1 Division of Pulmonary, Critical Care and Sleep Medicine, University of Cincinnati College of Medicine, Cincinnati, USA; 2 Department of Veterans Affairs, Cincinnati, VA Hospital, Cincinnati, USA

**Keywords:** Mechanisms of disease, Translational research

## Abstract

Chronic Obstructive Pulmonary Disease (COPD) is the third leading cause of death worldwide. COPD is frequently punctuated by acute exacerbations that are precipitated primarily by infections, which increase both morbidity and mortality and inflates healthcare costs. Despite the significance of exacerbations, little understanding of immune function in COPD exacerbations exists. Natural killer (NK) cells are important effectors of innate and adaptive immune responses to pathogens and NK cell function is altered in smokers and COPD. Using high-dimensional flow cytometry, we phenotyped peripheral blood NK cells from never smokers, smokers, and COPD patients and employed a non-supervised clustering algorithm to define and detect changes in NK cell populations. We identified greater than 1,000 unique NK cell subpopulations across patient groups and describe 13 altered NK populations in patients who experienced prior exacerbations. Based upon cluster sizes and associated fluorescence data, we generated a logistic regression model to predict patients with a history of exacerbations with high sensitivity and specificity. Moreover, highly enriched NK cell subpopulations implicated in the regression model exhibited enhanced effector functions as defined by *in vitro* cytotoxicity assays. These novel data reflect the effects of smoking and disease on peripheral blood NK cell phenotypes, provide insight into the potential immune pathophysiology of COPD exacerbations, and indicate that NK cell phenotyping may be a useful and biologically relevant marker to predict COPD exacerbations.

## Introduction

Chronic Obstructive Pulmonary Disease (COPD) is the third leading cause of death in the United States and worldwide^1^. Costs related to the treatment of COPD and associated comorbidities are a significant economic burden to patients and health care systems. Acute exacerbation in COPD (AECOPD) is a significant risk factor contributing to increased morbidity and mortality in affected COPD patients. Exacerbations may also result in significant reductions in lung function that is in part due to increased airway and systemic inflammation. Acute worsening of symptoms such as shortness of breath, increased cough, excessive mucus production, and enhanced fatigue often occur during COPD exacerbations. Additionally, an acute exacerbation can dramatically reduce a patient’s quality of life and significantly decrease lung function^2^. The initiators of exacerbations are still poorly understood but are likely due to inflammatory responses to bacterial or viral infections, or inhalation of environmental particulate matter. Destruction of lung parenchyma and tissue remodeling in COPD is likely due, in part, to pathological alterations in immune cell function^3^. Recent work has suggested that some patients may be more susceptible to exacerbations than others and represent an ‘exacerbator phenotype’ associated with COPD-dependent changes in systemic immune function^4^. Despite recent progress in defining the altered immune function in COPD, how smoking and COPD-related changes in immune function contribute to AECOPD remains poorly understood.

Natural killer cells are lymphocytes that function as part of the innate immune system and are now classified as part of the family of innate lymphoid cells 1 (ILC1)^5^. NK cells are well described in the context of host-rejection of virally infected cells and tumor surveillance. Surface receptors recognize tumor, virally infected, and stressed cells, as well as many pathogens. The activating (e.g. NKG2D, CD336, CD335) and inhibitory Killer-cell immunoglobulin-like receptors (KIR) receptors and their engagement with targets determine the cytokine and cytolytic response of NK cells. Activated cells release cytokines such as interferon gamma (IFNγ) and tumor necrosis factor alpha (TNFα), among others, which recruit and activate other immune system cells. Perforin release from NK cells causes pore formation in target cell membranes; proteases such as granzyme A and B can enter the cell to induce apoptosis by caspase cleavage as well as cleave other cellular targets. Therefore, aberrant and/or chronic activation of NK cells can result in chronic inflammation and tissue damage.

Given the morbidities and economic burden of COPD and AECOPD, it is important to examine the immune function of these patients in greater detail than previous studies. In this study, we utilized polychromatic flow cytometry to measure the expression of an array of activating and inhibitory receptors on peripheral blood natural killer cells from never smokers, current smokers, and COPD patients. We identified >1,000 significant and unique NK cell subpopulations using the Scalable Weighted Iterative Flow-clustering Technique (SWIFT)^6–8^. These data reveal several changes in the expression of NK cell activating/inhibitory receptors and the size of the NK cell subpopulations associated with smoking and COPD. Furthermore, we demonstrate that logistic regression analysis using population size and receptor expression from peripheral blood NK cells is highly predictive of patients with a previous exacerbation. These findings provide an entry point to more closely examine the effects of smoking on NK cell phenotype and function, the role of NK cells in COPD exacerbations, and the use of NK cells as biomarkers for exacerbations.

## Results

### Variability in surface expression of NK cell receptors in smokers and COPD patients

**Figure 1:**
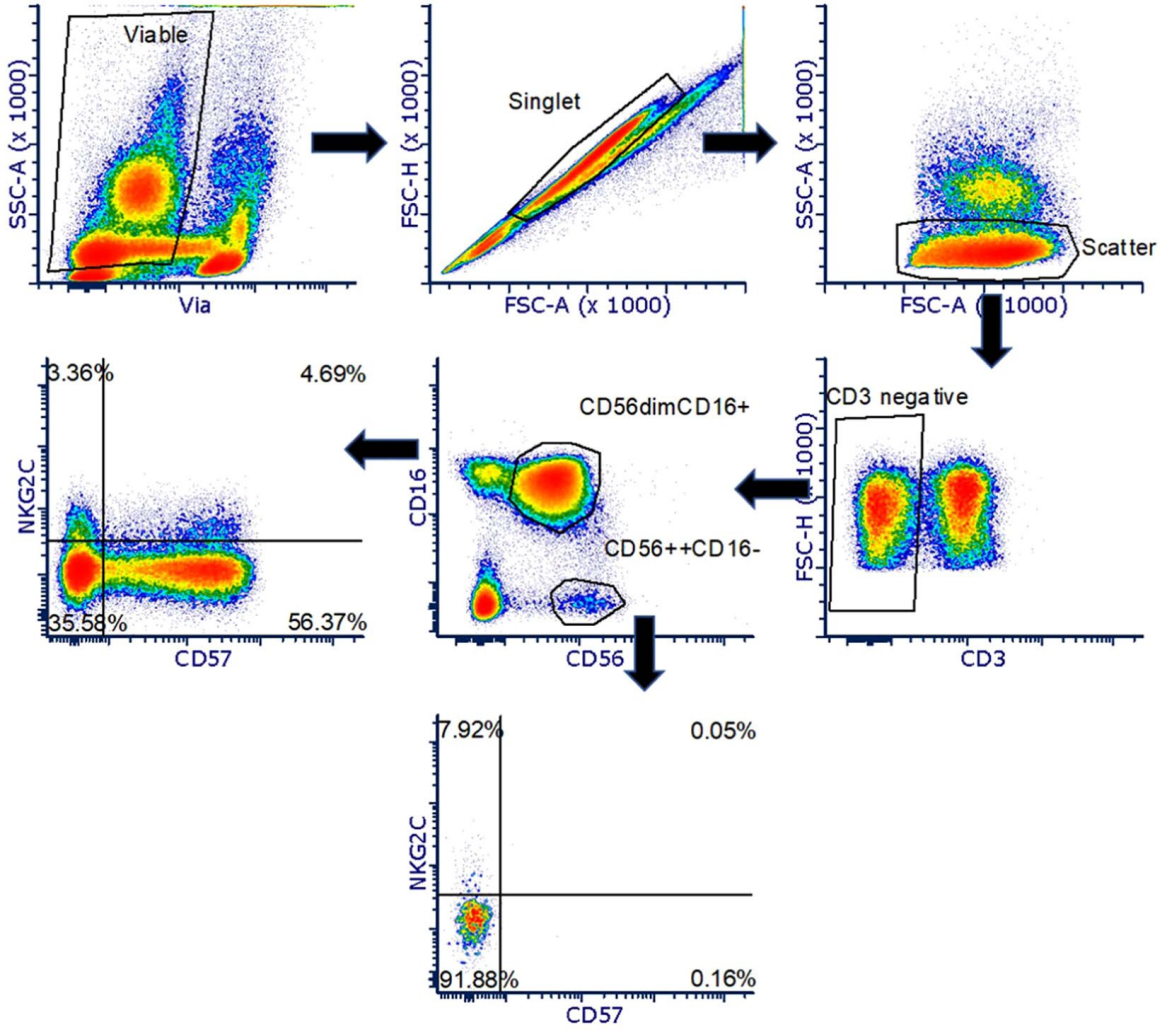
Representative gating strategy for NK cell phenotyping. Plots demonstrate the representative gating strategy for flow cytometry data to identify NK cells and the expression of activating and inhibitory receptors.

Given the recent discovery of a vast array of potential NK cell subpopulations by high resolution analyses^9^ and the likely influence of environmental factors in the shaping of the NK cell repertoire, we hypothesized that unique NK cell subsets emerge in response to long term smoke exposure and as a consequence of COPD exacerbations. To address this hypothesis, we analyzed NK cells from PBMC samples from 5 patient groups collected at the Cincinnati Veterans Administration Hospital. The patient groups included Never Smokers (NS), Current Smokers (CS), Former Smokers (FS), Gold I/II COPD, and Gold III/IV COPD. Patient demographic and spirometry data are shown in Table 1. All statistical comparisons are to the NS group. NK cells were first identified by flow cytometry as CD3− CD56+ CD16+ and activating/inhibitory expression was subsequently defined in the populations as shown in Fig. 1. Based on the finding that the expression of the activating receptors was the predominant distinguisher of the subtypes^9^, we developed a panel of 12 antibodies heavily weighted in activating receptors and activation markers to probe the differences in NK cell populations between these groups (CD3, CD16, CD56, CD335(NKp44), CD335(NKp46), KLRC1, NKG2C, CD314(NKG2D), CD57, CD69, and inhibitory receptors CD158a, CD158b, along with a live/dead dye).

**Table 1:** Demographics of patient population.

	Never Smoker (NS)	Current Smoker w/o COPD (CS)	Former Smoker w/o COPD (FS)	Gold I/II	Gold III/IV
Age (yrs)	58+/−4.22	56.6+/−3.3	58.6+/−3.63	64.3+/−2.66	68.1+/−2.01
Sex (M/F)	14/0	9/3	12/0	10/0	9/0
Pack Years	0+/−0	32.1+/−5.9*	43.6+/−11.5*	49.4+/−8.3*	53.4+/−10.1*
Pre FVC (L)	3.7+/−0.3	3.4+/−0.2	3.7+/−0.2	3.7+/−0.2	2.2+/−0.1*
Pre FEV_1_ (L)	3.0+/−0.2	2.7+/−0.2	2.7+/−0.1	2.3+/−0.1	1.0+/−0.1*
Pre FEV_1_/FVC%	81.4+/−2.1	81.3+/−1.3	74.4+/−1.5*	64.3+/−2.6*	50.3+/−3.5*
Post FVC (L)	3.71+/−0.3	3.56+/−0.2	3.75+/−0.2	3.96+/−0.2	2.47+/−0.2*
Post FEV_1_ (L)	3.14+/−0.2	2.93+/−0.2	2.87+/−0.1	2.43+/−0.1	1.2+/−0.1*
Post FEV_1_% Predicted	87+/−3.7	93.8+/−4.6	88.0+/−6.3	72.8+/−4.7*	38.2+/−3.6*
Post FEV_1_/FVC%	84.5+/−1.8	82.6+/−1.6	76.8+/−1.6*	61.4+/−1.5*	49.1+/−3.6*
Total Exacerbations (n), last 2 years	2	2	1	5	13
Average Exacerbations per patient, last 2 years	0.11+/−0.11	0.17+/−0.11	0.1+/−0.10	0.5+/−0.31	1.45+/−0.39*
Corticosteroids use (n)	2/14	5/12	1/12	3/10	8/9

**Figure 2:**
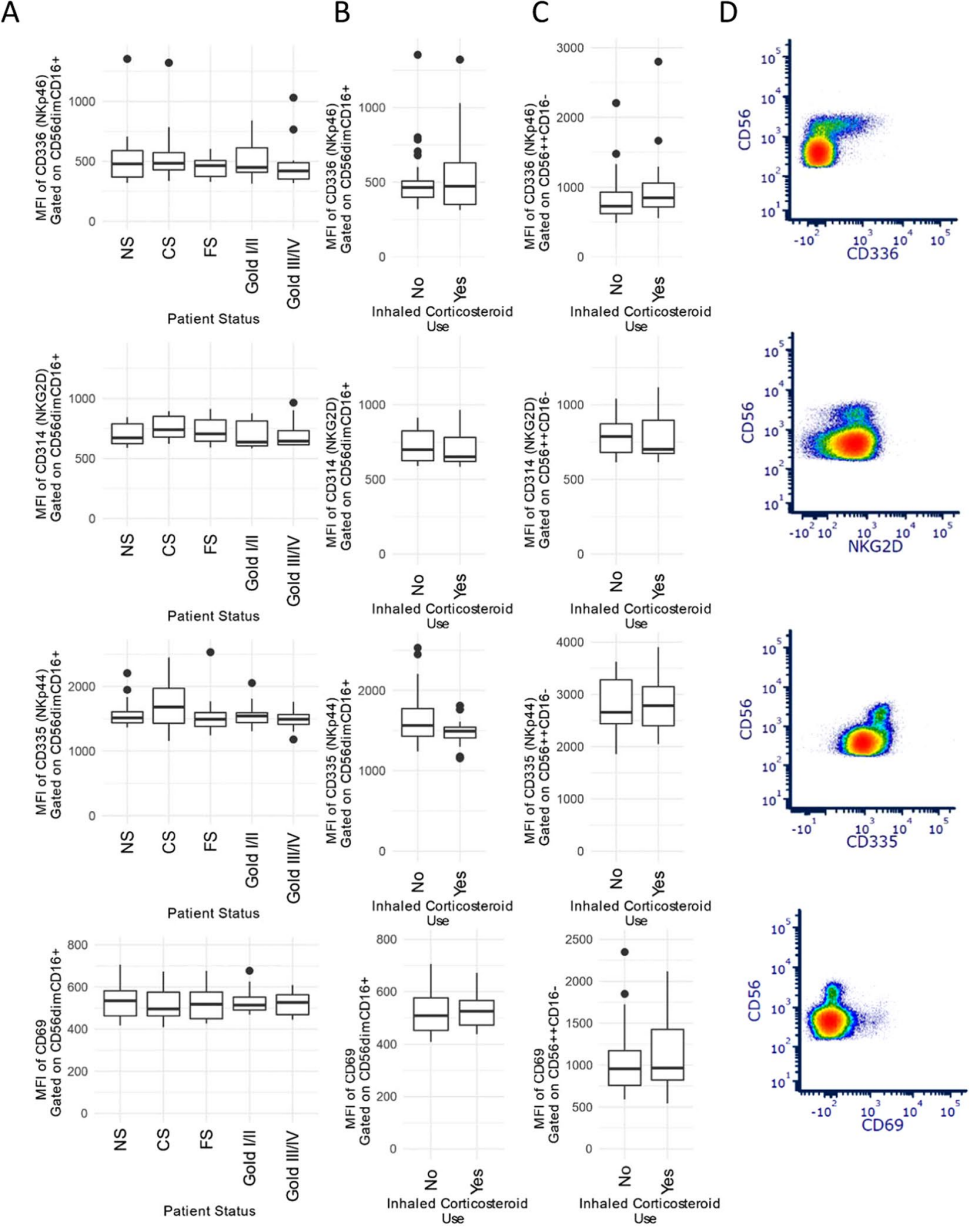
NK cell surface activating receptor expression in patient groups. The median fluorescence intensity (MFI) of the surface receptors are shown by smoking and COPD status. (**A**) The data show fluorescence of CD336, CD314, and CD335 based on COPD status of CD56dimCD16+ NK cells. Each patient group is represented by a boxplot that shows the median and interquartile range. (B) The effects of a prior inhaled corticosteroid (ICS) administration on CD336, CD314, and CD335 are shown for CD56dimCD16+ NK cells. The ICS use was, due to exclusion criteria, more than one month before enrollment into the study. (**C**) The effects of inhaled corticosteroids on CD56 ++ CD16− NK cells are shown. (**D**) representative scatter plots of CD336, CD314(NKG2D), CD69, and CD335 vs CD56.

**Figure 3:**
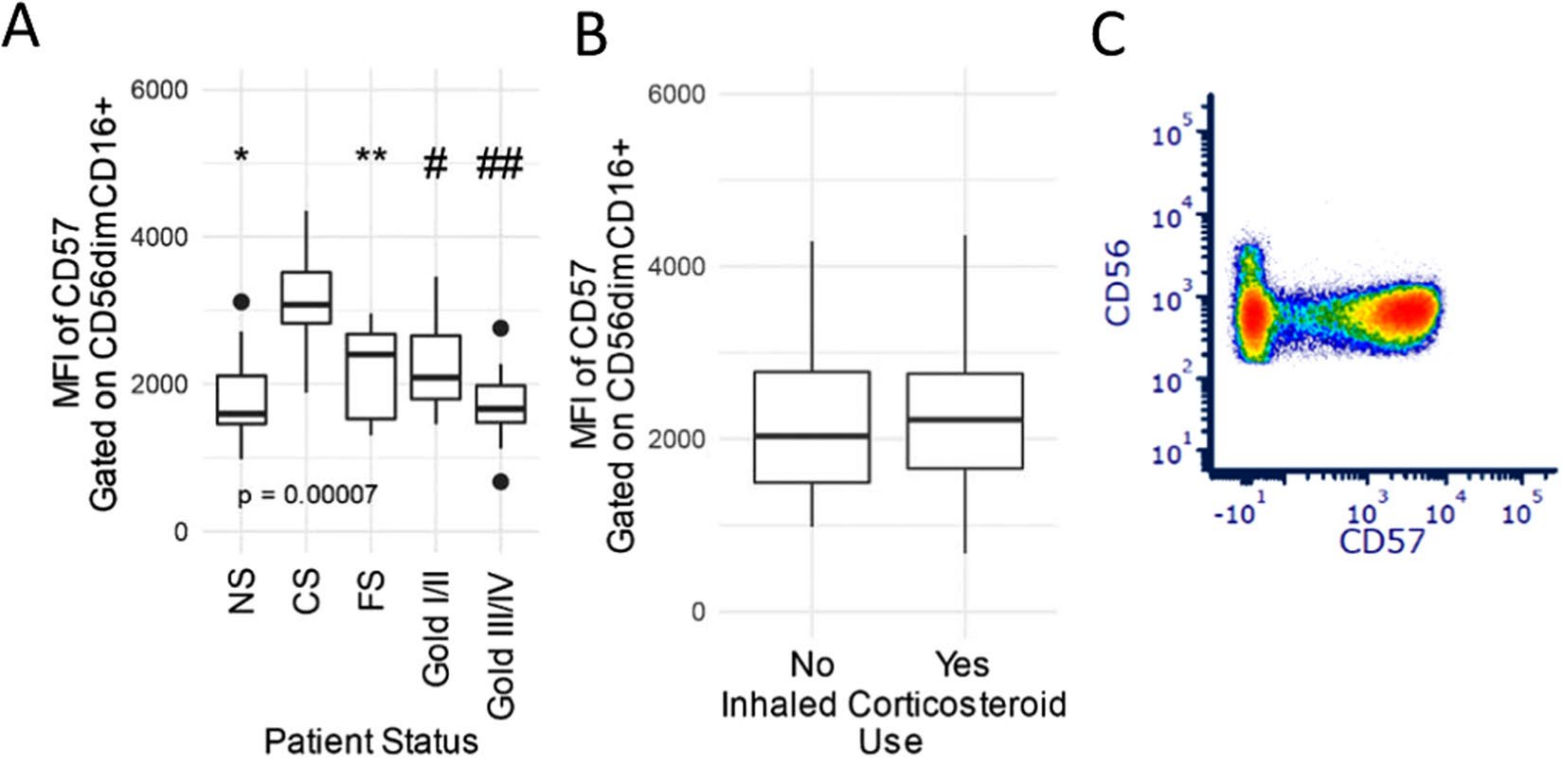
Bi-phasic NK cell CD57 expression and COPD disease progression. (**A**) Data indicates differences (p < 0.00007) between patient COPD groups and CD57 MFI on CD56dimCD16+ NK cells. (**B**) The effects of a prior inhaled corticosteroid (ICS) administration on CD57 are shown for CD56dimCD16+ NK cells. The ICS use was, due to exclusion criteria, more than one month before enrollment into the study. Data are represented by boxplots which show interquartile range (IQR); whiskers represent 1.5 × IQR. Data points beyond the whiskers are considered outliers. ANOVA comparisons of groups p = 0.00007, and post-hoc comparisons: *p = 0.00001 NS vs CS, **FS vs CS p = 0.006, # Gold I/II vs CS p = 0.003, ## Gold III/IV vs CS p = 0.0001 (**C**) Representative scatter plots of CD57 and CD56.

The data reveal few significant effects of smoking or COPD status on the mean fluorescence intensity (MFI) across the entire population of NK cells. The expression of CD336 (NKp46), CD314 (NKG2D), and CD335 (NKp44) are shown in Fig. 2A. Inhaled corticosteroids (ICS) are potent immunomodulators and high doses of ICS are routinely administered in response to COPD exacerbations. Indeed, high doses of ICS use has been shown, both *in vivo* and *in vitro*, to be associated with alterations to NK surface phenotype and function^10,11^. Therefore, patients with an exacerbation and possible ICS use in the month prior to enrollment were excluded. We examined the effects of routine, maintenance dose ICS on surface NK cell receptor expression in the two primary NK cell populations. Figures 2B,C demonstrate there are no significant effects of ICS in either CD56^dim^CD16^+^ or CD56^+^CD16^−^ NK cells. Representative scatter plots are shown in Fig. 2D. Interestingly, we did observe differential CD57 expression across COPD groups. Current smokers demonstrated the highest expression of CD57 which appears to decline with increased severity of COPD (Fig. 3B). As with other markers, we did not observe any difference between CD57 due to ICS use (Fig. 3B). Representative scatter plots are shown in Fig. 3C.

### High-dimensional analysis of NK cell receptor expression in unique NK cell subpopulations

**Figure 4:**
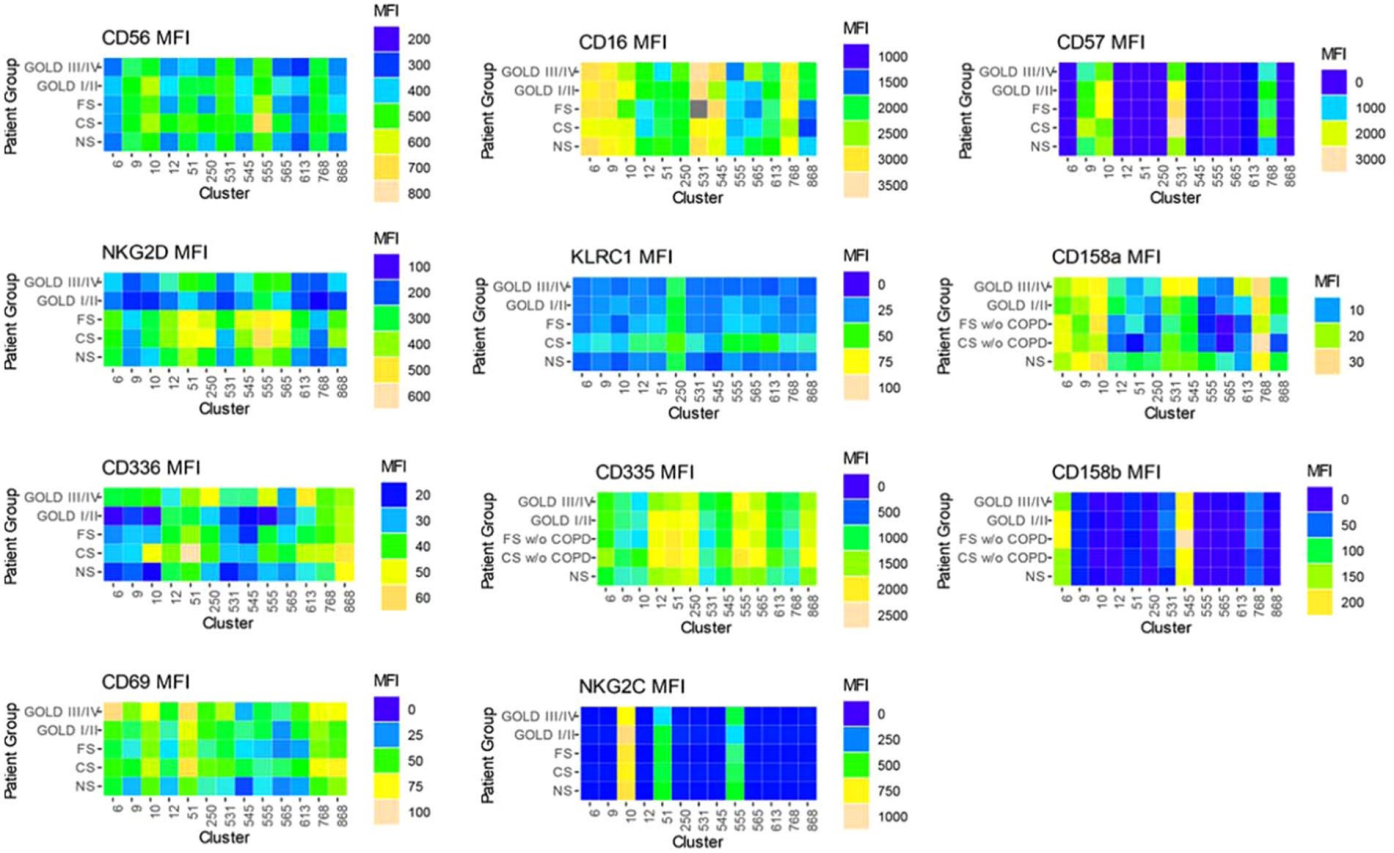
SWIFT clustering reveals variable surface expression of activation markers across NK cell subpopulations. Median fluorescence intensity of the significant CD56+ SWIFT clusters. Differences in expression levels for each receptor are easily visualized between patient groups (columns) and NK cell subpopulations (rows). The average color value indicates the mean of the median fluorescence of each cluster, for each marker, and each patient group (n = 9–14 patients per group).

Polychromatic flow cytometry experiments have increasing analysis complexity as parameters increase. Two by two scatterplot comparisons of fluorescent parameters may not show complex relationships between surface markers and these cell phenotypes may be missed using a manual gating strategy. Manual analysis is also subject to bias and subjectivity in setting gates^12^. Therefore, we employed a non-supervised clustering algorithm to analyze NK cell phenotypes. The SWIFT (Scalable Weighted Iterative Flow-clustering Technique) algorithm was used to analyze our data as this algorithm preserves important biological subpopulations in data from large high dimensional data sets and is capable of detecting rare subpopulations^7^. Briefly, SWIFT is a mixture model clustering that first identifies all clusters present within the data by patient group (i.e NS, CS, FS, Gold I/II, Gold III/IV) which generates a “template” cluster description. The “templates” are then combined into a joint model and then clusters identified in individual patient data files. For each cluster present, cells compete for membership in the identified clusters. This process serves to identify subsets of cells that are altered between patient groups. SWIFT clustering analysis identified 1041 cell clusters across the five patient groups. To limit the number of clusters with few cells we took the average cluster size per patient group, then summed across COPD groups and discarded clusters with less than 1000 cells across the 5 patient groups. From these clusters we identified 28 clusters with significant changes by performing Kruskal-Wallis one-way analysis of variance followed by a 10% false discovery rate (FDR) multiple test correction. These clusters were further restricted to 13 clusters that were CD56^+^ representing NK cells from peripheral blood. These analyses indicate that smokers and COPD patients exhibit unique NK cell populations marked primarily by lower CD56 expression, high CD57 expression (activation and maturity marker), and high expression activation markers NKp46 and NKp44 (Fig. 4).

### NK cell subpopulation sizes are associated with smoking, disease severity and exacerbations

**Figure 5:**
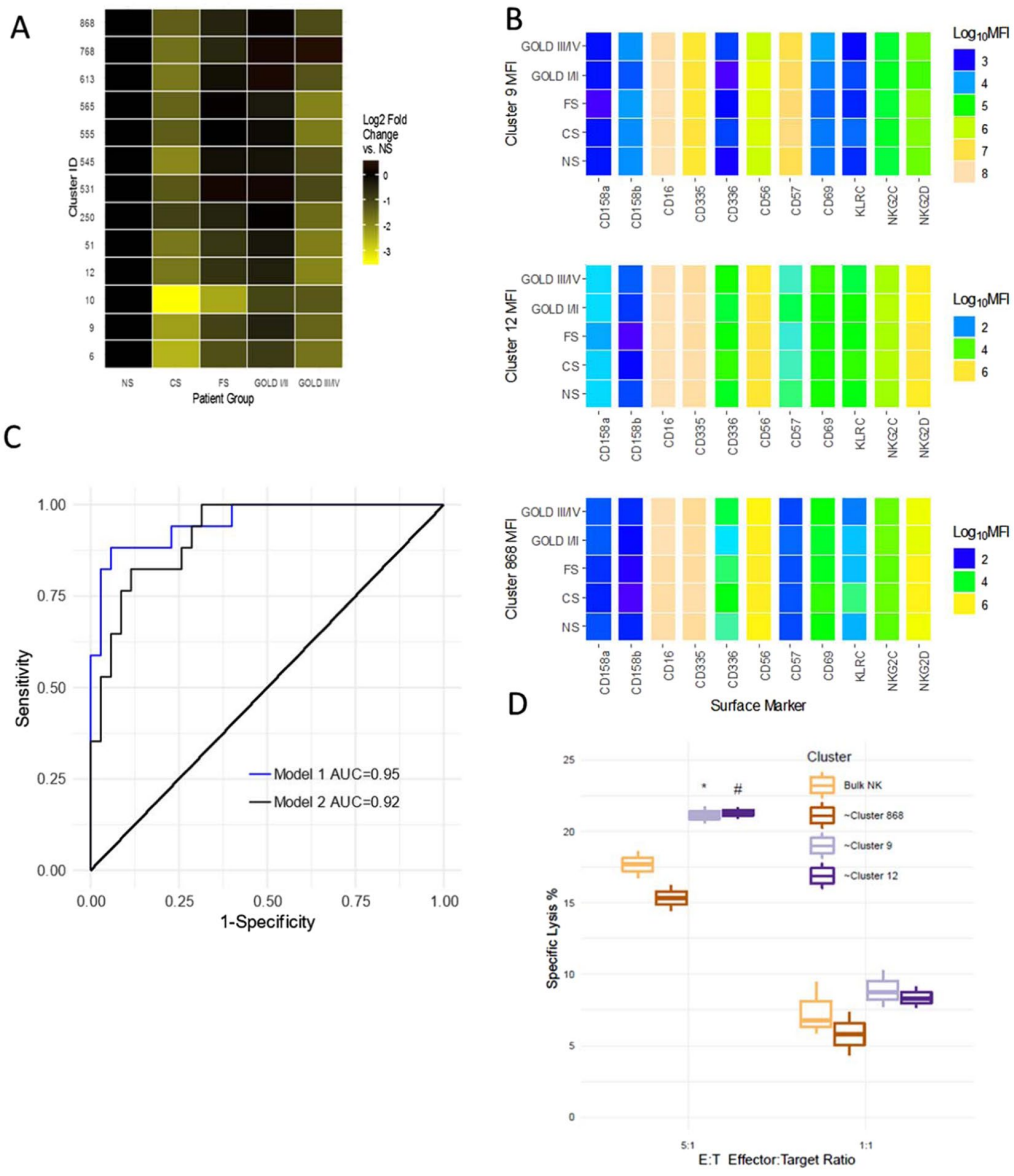
Logistic regression of cluster size reveals NK clusters are markers for prior exacerbations. (**A**) The significant SWIFT clusters after a false discovery rate correction clusters are shown as log2-fold change vs the Never Smokers. All clusters shown are from Viable, CD3−, CD56+ cells. The intensity of the yellow indicates the change in cluster size of that cluster in that patient population. (**B**) Color heat maps of fluorescent parameters of the clusters used in the logistic regression. (**C**) Receiver Operator Curves for model 1 and 2 of the logistic regressions with exacerbation as outcome variable. Model 1 includes spirometry data and cluster size and fluorescent data of clusters 9, 12, 868. Model 1 has an AUC of 0.95. Model 2 represents a logistic regression that only includes cluster sizes and fluorescence data. Model 2 has an AUC of 0.92. (**D**) Magnetic bead enriched CD56+ cells from 3 healthy never smokers were sorted into 4 populations, bulk NK, cluster 9, cluster 12, and cluster 868. Appropriate quantities (25,000 or 5,000) of NK cells were incubated with 5,000 K562 cells to measure cytotoxicity. NK cells and target cells were incubated with IL-2 (100 U/ml) for 4 hours at 37C in 5% CO2. Subsequently, cells were stained with propidium iodide (5 ug/ml) and dead and live cells measured by flow cytometry. % Specific lysis is shown. The overall 2-wayANOVA for the data shows that both the cytotoxicity due to E:T ratio and Clusters are significant, P < 0.000 and P < 0.001 respectively. Specific Post-hoc contrasts indicate that cluster 9 and 12 are increased compared to bulk NK cells, *P < 0.020 and ^#^P < 0.017.

Although high-dimensional analysis is informative in terms of receptor expression levels and identifying potentially important subpopulations, it is equally powerful in describing the changes in the sizes of the subpopulations. Interestingly, we observed several decreases in the relative abundance of these 13 NK cell subpopulations as a consequence of smoking and disease severity. Figure 5A demonstrates the change in NK cell population cluster sizes and shows a generalized trend for decreased population sizes in smokers and GOLD III/IV patients compared to never smokers. Similar to the observations in the bulk analysis of NK cell surface receptor expression, we observed few differences on the unique subpopulations between patient groups for individual markers (Fig. 5B). Representative results are shown for clusters 9, 12, and 668. Surprisingly, however, these analyses did reveal several-fold differences in expression of individual NK cell receptors between the subpopulations which is most clearly visualized by comparing CD57 expression between the subpopulations (Fig. 5B).

Finally, we examined the relationship between NK cell subpopulations and COPD exacerbations using the significant SWIFT clusters as predictors a prior of exacerbations as the outcome in a logistic regression. SWIFT cluster sizes and the associated cluster fluorescence from the 13 clusters after FDR and CD56^+^ selection for dependent variables in the logistic regression. In addition, we included spirometry data in the regression. Out of the initial 13 clusters, only 3 were statistically significant contributors to the logistic regressions. The resulting logistic regression (model 1) includes the size of three specific clusters (Fig. 5A, Cluster 9, 12, 668) as well as some of the NK surface makers specific to those clusters (Fig. 5B), and Pre and Post FEV_1_, and FEV_1_/FVC%. The overall p-value for the model is p = 0.00001. The pseudo-R2 Nagelkerke is 0.737 and overdispersion is 0.634. Using this model, the receiver operator curve (ROC) has an area under the curve (AUC) of 95% (Fig. 5C). We also performed an additional logistic regression model (model 2) that did not include spirometry data. This model only includes SWIFT cluster and fluorescent data. The overall p-value is 0.000074, and the Nagelkerke pseudo R2 is 0.58, and overdispersion 0.827. The AUC in the ROC for this model is 92% (Fig. 5C). The addition of the spirometry data provides modest additional sensitivity/specificity in the ROC. Taken together these data indicate that exacerbation risk can be modeled by distinct clusters of natural killer cells found in peripheral blood of patients. The most well defined effector function of NK cells is the innate capacity to directly kill foreign cells. To examine potential functional differences between these unique populations, we determined if bulk NK cells and NK cells approximating clusters 9, 12, and 868 were differentially cytotoxic. These studies reveal increased cytotoxicity effector function of cluster 9 and cluster 12 compared to bulk NK suggesting a potential biological link between their diminished population sizes and susceptibility to exacerbations.(Fig. 5D).

## Discussion

In humans, two NK cell subsets have been well characterized: CD56^dim^CD16^+^ NK cells which are considered the mature NK subset and CD56^bright^CD16^neg/dim^ NK cells which are considered immature^13^. CD56^dim^CD16^+^ NK cells generally produce low levels of cytokine and exhibit high levels of cytotoxicity whereas CD56^bright^CD16^neg/dim^ NK cells produce high cytokine levels and exhibit low cytotoxicity. These effector functions are regulated by an array of activating and inhibitory surface receptors that can recognize tumor, virally infected, and stressed cells, as well as many pathogens. The activating (e.g. NKG2A, NKG2C, NKG2D) and inhibitory Killer-cell immunoglobulin-like receptors (KIR) receptors can define subsets of NK cells and their engagement with targets ultimately determine the cytokine and cytolytic responses of NK cells. Although CD56/CD16 markers are broadly utilized to identify NK cells, changes in expression levels of specific surface markers can also be used to define specific subsets in health and disease. For example, a unique subset of NK cells that express high levels of CD57 and NKG2C expands during human cytomegalovirus (CMV) infection^14^. This subpopulation exists across species as a very similar subset of NK cells expands and persists in mice infected with CMV^15^. Another example of functional division of NK cell subsets is achieved by CD27 and CD11b expression levels to discriminate maturation levels^16^. Characterizing NK cells from distinct tissues and during infections have also revealed specialized subsets that reflect inherent cellular plasticity in terms of activation and utilization of various signaling pathways^17,18^.

In addition to providing insight into the population level biology of exacerbations, our analysis provides insight into individual markers used to generate the population identifications. For example, the activation/NK cell memory marker CD57 is expressed at significantly higher levels in smokers, former smokers, and GOLD I/II COPD patients compared to never smokers. CD57 expression is reported to be a marker of mature and ‘memory’ NK cell and is found within the more mature CD16^+^CD56^dim^ population of NK cells^14^. Functionally, CD57 is associated with cell adhesion and homing to inflamed tissue. Increased CD57 is also associated with cytomegalovirus (CMV), or Epstein-Barr virus (EBV) infection. The reason for the increased CD57 expression in current smokers is unknown. One plausible explanation is the increased inflammatory signaling milieu due to cigarette smoke. We have previously shown that CS exposure primes and enhances NK activation in mice^19^ and NK cell function is long understood to be altered in smokers^20^. This suggests that smoking-dependent chronic inflammation may drive the activation and maturation of CD16^+^CD56^dim^ NK cells due to the inflammatory milieu^21^. We also observed a decline in CD57 MFI as COPD disease progression increases. This finding seemingly contradicts previous studies by Olloquequi *et al*. that show increased CD57+ cell density in pulmonary connective tissue and increased density of CD57+ cells in pulmonary lymphoid follicles of late stage COPD patients^22,23^. However, our study examines NK cells in the peripheral blood whereas other studies examined CD57+ cell by immunostaining preserved lung tissues without discriminating the cell type expressing CD57. Furthermore, an important physiologically role of CD57 is adhesion and homing. Therefore, our data may be reflective of these observations in lung tissue whereby increased CD57 expression results in increased migration from the periphery into tissues.

It is important to emphasize that although there are significant changes in surface expression of NK cell receptors, the majority of changes in significant SWIFT NK populations are based on the decreasing size of the population. This infers that the populations are contracting as a consequence of smoking/COPD/exacerbations or these patient groups inherently possess fewer of these NK cell subpopulations. That said, there are several potential outcomes to these studies that we can anticipate based on the data and published literature. One possible outcome is that the identified subpopulations are equally responsive on a per cell basis but the decreased numbers of these cells in exacerbators render the NK cell pool less responsive to challenge. Another possibility is that these subpopulations are functionally repressed which, together with fewer numbers of cells, increases the susceptibility to exacerbation. These would be straightforward conclusions and easily interpreted from the results. However, we hypothesize that the results present a more complex picture of NK cell effector function. Previous studies have demonstrated that there are distinct differences in NK cell subpopulations based on CD56 expression. Moreover, high CD57 expression (which we observe in smokers and COPD patients) is associated with increased cytotoxicity and lower cytokine responsiveness representing a more mature phenotype and demarcate NK cells associated with memory against prior CMV infection^14^. However, we see both increased cytokine responsiveness and cytotoxicity in smokers/COPD patients. These findings do not align with the proposed singular functions of these expression levels and suggests we are identifying unique populations of NK cells in the context of COPD.

Potentially, the changes in observed NK phenotypes may be due to ICS use in COPD patients rather than exacerbations. We examined NK surface phenotype in the study patients in relation to ICS use and did not observe any ICS dependent changes in surface phenotype. Van Ierssel *et al*. report that budesonide or prednisolone treatment decreased NK cell activity in Crohn’s disease but by 10 weeks post-steroid treatment, NK activity had returned to baseline in the budesonide group and was enhanced in the prednisolone group^24^. Systemic hydrocortisone treatment results in significant changes in immune function. However, by 7–28 days post steroid treatment cell phenotype and function had largely returned to baseline^25^. The patients in our study were recruited from a population of VA patients who had at no exacerbations in the month prior to enrollment. Taken together, it is likely that most exacerbators were not currently on ICS at the time of sample collection.

Classification of COPD exacerbation risk is an important metric in the care of COPD patients. Exacerbations increase morbidity and mortality in these patients as well as augment health care costs and resource utilization. As opposed to the subjective and variable physical and physiological endpoints used to guide therapy in stable COPD, there are no reliable indicators of a patient’s exacerbation status, susceptibility, or disease progression. Accordingly, there is a growing interest in the development and use of biomarkers to assess disease and predict disease progression in COPD^26–29^. Ideally, biomarkers will better reflect disease activity while representing biologically relevant pathways. Theoretically, biomarkers of COPD exacerbations will identify subgroups of patients who would benefit from specific interventions and conversely avoid the side effects of prolonged exposure to unnecessary drugs. Furthermore, the identification of biomarkers associated with COPD exacerbations will undoubtedly guide future basic research into the mechanisms contributing to the pathogenesis of COPD leading to new therapeutic targets. There are few biological markers that correlate to exacerbation risk or susceptibility except having had a prior exacerbation^30,31^. One natural candidate for increased exacerbation risk, or an “exacerbator phenotype”, is the altered immune system of COPD patients. Our data clearly demonstrate there are long lasting alterations to NK cell subpopulations in COPD patients compared to NS and that these changes are associated with prior exacerbations. Thirteen CD3−CD56+ clusters were significantly altered and three of these were strong predictors of a prior exacerbation by logistic regression. Our data indicate that a patient who has had a prior exacerbation subsequently has an identifiable change in natural killer cell phenotype in their peripheral blood. We cannot determine if the changes we observe in NK populations precede or are in response to an exacerbation. These cells may represent a susceptible phenotype of NK cells that preferentially undergo apoptosis in response to an exacerbation^32^. Alternatively, the decline in cluster size may represent a change in surface phenotype, and therefore cluster membership, that our analysis cannot track. The COPD and smoke-dependent cytokine milieu may shape the development and maintenance of NK cell clusters. Two clusters (9 and 12) had moderate to high CD57 expression indicating that they are composed of mature, terminally differentiated NK cells. The observed differences in cytotoxicity between clusters 9 and 12 compared to bulk NK cells imparts plausible biological relevance as well. This finding suggests that these populations of cells may be more responsive to stimuli associated with exacerbations and consistent with our observations that the sizes of these clusters are decreased in patients with prior exacerbations. Other work has shown that there is an increase in CD57+ cells in COPD lungs. Therefore, the reduction in cluster size is potentially due to migration into tissue from the periphery. The phenotypes of these clusters may be a biomarker of exacerbation susceptibility and/or also represent an ‘exacerbator phenotype’. Taken together, cigarette smoke causes changes in an NK cell phenotype profile that is stable across patients with differing levels of COPD severity stratified by airflow severity.

Our patient samples only include a sample of whole blood and spirometry taken at the time of study enrollment. Demographic data and the number of exacerbations in the prior two years were recorded at study enrollment. Additionally, our exclusion criteria rejected patients with an exacerbation in the month prior to enrollment. Therefore, these clusters and associated cluster phenotypes are stable for up to two years and the changes in phenotype represent long-term alterations in the immune profile of COPD patients with and without exacerbations. If the NK phenotype is present prior to an exacerbation, perhaps due to cigarette smoke exposure, COPD state, and/or genetic background, then these populations may be biomarkers of exacerbation risk. Alternatively, if these NK phenotypic changes occur in response to or after an exacerbation, they provide a window to study altered immune responses in patients who are now at increased risk of a future exacerbation^30^. Our analysis is partly limited due to the relatively small sample size. As a result, we were unable to split the data into training and test data sets to validate the logistic models. Additionally, the surface markers we used may not have the depth to completely discriminate populations of NK cells in peripheral blood. While our data show a relation between exacerbation and peripheral blood NK phenotype, we were unable to determine exacerbation cause (e.g. bacterial, viral, or environmental).

In summary, we assessed the expression of an array of activating and inhibitory receptors on peripheral blood NK cells from never smokers, current smokers, and COPD patients using flow cytometry and high-dimensional analyses. These data showed few significant changes in the expression of NK cell receptors within very large classifications of NK cells but reveal several changes in i) the expression of NK cell activating/inhibitory receptors among NK cell subpopulations, and ii) the size of the NK cell subpopulations associated with smoking and COPD. Furthermore, we demonstrate that logistic regression analysis using population size and receptor expression from peripheral blood NK cells is highly predictive of patients with a previous exacerbation. These findings provide an entry point to more closely examine the effects of smoking on NK cell phenotype and function, the role of NK cells in COPD exacerbations, and the use of NK cells as biomarkers for exacerbations. Future separate studies that more comprehensively probe the many effector functions of the various populations of NK cells will help to define these novel biomarkers and immune pathways that can be used therapeutically to manage COPD exacerbations and slow the progression of this disease.

## Methods

### Patients and sample collection

Post bronchodilator spirometry was performed on enrolled patients. Whole blood was withdrawn by venipuncture into anti-coagulated lithium heparin tubes and immediately processed to isolate peripheral blood mononuclear cells (PBMC) by Lymphoprep density gradient centrifugation using SepMate-50 tubes (Stemcell Technologies). PBMC were then cryo-preserved in freezing media (50% RPMI 1640, 40% FBS, 10% DMSO) in a Mr. Frosty Freezing Container (ThermoFisher) and then stored in liquid nitrogen until use. At the initiation of this study the GOLD I-IV staging was standard of practice at the Veterans Administration hospital in Cincinnati^33^. During the course of this work a new classification system for COPD patients became more widely employed. For consistency, we have not adopted the 2017 guidelines for COPD staging, and retain the Gold I-IV staging based on FEV1% predicted^34^. All methods were performed in accordance with the relevant guidelines and regulations.

### Flow cytometry

NK cell surface staining was performed on quick-thawed peripheral blood mononuclear cells (PBMC). Antibodies for flow cytometry were obtained from BD Biosciences (San Jose, CA): BV786 mouse anti-human CD3 (SK7); BUV395 mouse anti-human CD56 (NCAM16.2); BUV737 mouse anti-human CD16 (3G8); R-PE mouse anti-human NKp44/CD336 (p44–8); BV510 mouse anti-human NKp46/CD335 (9E2/NKp46); BV650 mouse anti-human NKG2D/CD314 (1D11); BV711 mouse anti-human CD158a (HP-3E4);. Antibodies for FITC mouse anti-human NKG2C/CD159c (134591) were obtained from Biotechne (Minneapolis, MN). Antibodies for R-PE-Cy7 mouse anti-human CD158b (DX27) and mouse anti-human CD57 (HNK-1) were purchased from BioLegend (San Diego, CA). PerCP-efluor 710 mouse anti-human CD69 (FN50) and LIVE/DEAD Fixable Near-IR Dead Cell stain kit were purchased from ThermoFisher Scientific.

Briefly, cells were thawed and washed in ice cold media (RPMI 1640, phenol red free) labelled with Fixable Near-IR Live/Dead viability dye for 20 minutes on ice and in the dark. Cells were then washed in ice-cold 2X in flow buffer (FB, 1X PBS, 0.1% sodium azide, 0.5% bovine serum albumin, pH 7.4) to remove excess Live/Dead viability dye. Cells were then blocked with Fc Receptor Binding Inhibitor (ThermoFisher, San Diego, CA) and mouse γ-globulins (Sigma-Aldrich, St Louis, MO) for 1 hour in the dark at 4 °C. Cells were then stained with appropriate antibodies for 1 hour at 4 °C in the dark. Cells were then washed 2X in FB and immediately analyzed by flow cytometry (BD Bioscience). Batches of samples (n = 6–7) were analyzed at a time. All flow cytometric data were acquired using equipment maintained by the Research Flow Cytometry Core in the Division of Rheumatology at Cincinnati Children’s Hospital Medical Center. Samples were analyzed on a 5-laser BD Fortessa 2 equipped with 355 nm (UV), 405 nm (violet), 488 nm (blue), 561 nm (yellow/green) and 640 nm (red) lasers that allow for the detection of 2 scatter parameters and up to 18 fluorescence parameters. Appropriate filters were used to collect data from the fluorophores used in the study. Data was collected with Diva 6.02 and analyzed with FCS Express V5 (De Novo Software, Glendale, CA). Cells were gated first on the lymphocyte population based on forward scatter area (FSC-A) vs. side scatter area (SSC-A) scatter. Next, doublets were excluded by gating (FSC-A vs. FSC-H) on single cells. Subsequently, cells were gated on viable cells. Where necessary CD3^−^ negative cells were then gated and NK cells were discriminated based on CD16^++^ CD56^dim^ vs. CD16^−^ CD56^bright^. Appropriate negative controls and fluorescence minus one (FMO) were used to set gates for the above populations. UltraComp eBeads (eBioscience) were used to determine the compensation between fluorophores.

### SWIFT clustering

Scalable Weighted Iterative Flow-Clustering (SWIFT) clustering was used to identify unique peripheral blood phenotypes unique to VA COPD patient groups^6–8^. SWIFT 3.0 scripts were used in MATLAB (R2016a) to generate the cluster profiles. Briefly, new concatenated fcs files were created by subsampling the individual patient fcs files within each patient group. The fcs files were first gated on viable and CD3- cells and then used in the SWIFT analysis. The data for each fluorophore was arcSinh transformed and then each consensus patient group fcs files (NS, CS, FS, Gold I/II, Gold III/IV) were run through the main SWIFT clustering algorithm. The resultant cluster template profiles were then combined, and the individual patient fcs files run through the joint template data. The output from this analysis included comma separated values (csv) files with the cluster ID as well as the number of cells in each cluster in each specimen. We limited the clusters we examined in by selecting clusters with at least 1000 cells across treatments, or approximately 1% of the CD56+ cells collected from each patient. SWIFT identified 1041 clusters, and after selection of clusters with >1000 cells, and a 10% FDR we were left with 17 clusters. Of these, 13 clusters were CD56+ NK cells.

### Cytotoxicity

Frozen PBMC from three never smokers were quickly thawed and NK cells isolated with anti-CD56 magnetic beads (Miltenyi) according to manufacturer’s instructions. Enriched NK cells were incubated with Alexa Fluor 647 anti-human CD57 (HNK-1), BV650 mouse anti-human NKG2D/CD314 (1D11); and PerCP-efluor 710 mouse anti-human CD69 (FN50) for 20 min, on ice, in the dark. Cells were washed with IMDM with 10% FBS and placed on ice for sorting. Cells were sorted on a MoFlo XDP (Beckman Coulter) at the Cincinnati Children’s Hospital Research Flow Cytometry Core. Cells were sorted into 4 populations that approximate bulk NK cells and implicated clusters: Cluster 12, CD57 + NKG2D + CD69+; Cluster 9, CD57 + NKG2D + CD69-; Cluster 868, CD57-NKG2D + CD69− and bulk NK cells, CD57− NKG2D + CD69-. Cells were placed in IMDM with 10% FBS and incubated with IL-2 (100 U/ml) in a cell culture (37 C, 5% CO2) incubator for 1 hour. An appropriate quantity of sorted NK cells was combined with 5,000 K562 target cells in round bottom 96-well plates in IMDM with 10% FBS and 100 U/ml IL2. Microwell plates were briefly centrifuged (3 min @ 300 g) to collect cells and then placed in a cell culture incubator for 4 hours. Cells were then removed and placed on ice for analysis by flow cytometry. Propidium Iodide (BioLegend) was added at a final concentration of 5ug/ml. Samples were then analyzed on an Attune Flow Cytometer (Invitrogen) and PI negative and positive K562 cells enumerated. Specific Lysis was determined as; (%PI positive cells - % PI positive background)/(100- % PI positive background).

### Statistics

All statistical tests were performed in R (3.3.2) and Rstudio (v1.0.153). Plots were generated using ggplot2 (v2.1). Differences between groups were considered statistically significant when p < 0.05. Analysis of Variance (ANOVA) and post-hoc tests were performed in R. Significant clusters were determined by using the non-parametric Kruskal–Wallis one-way analysis of variance test. Significant clusters with at least 1000 cells across the patient groups were then selected by employing a 10% False Discovery Rate (FDR). Logistic regression analysis employed generalized linear model component in R. Receiver Operator Curves (ROC) (v1.0.7) employed the ROCR package.

### Study approval

Study procedures were performed after obtaining written informed consent from patients visiting the Cincinnati Veterans Administration hospital. The study design was reviewed and approved by the VA Research and Development Committee and the University of Cincinnati Institutional Review Board (IRB# 2014–2354). All methods were performed in accordance with the relevant guidelines and regulations. Data from patients, such as questionnaires, spirometry results, and all specimens were assigned a unique study number to de-identify patient personal information.
